# Internists’ and intensivists’ roles in intensive care admission decisions: a qualitative study

**DOI:** 10.1186/s12913-018-3438-6

**Published:** 2018-08-08

**Authors:** Stéphane Cullati, Patricia Hudelson, Bara Ricou, Mathieu Nendaz, Thomas V. Perneger, Monica Escher

**Affiliations:** 1 Pain and Palliative Care Consultation, Division of Clinical Pharmacology and Toxicology, University Hospitals of Geneva, Rue Gabrielle-Perret-Gentil 4, 1211 Geneva, Switzerland; 2 Department of General Internal Medicine, Rehabilitation and Geriatrics, University of Geneva, Geneva, Switzerland; 3 Department of Community Medicine, Primary Care and Emergency Medicine, University Hospitals of Geneva, Geneva, Switzerland; 4 Intensive Care Unit, Department of Anaesthesiology, Pharmacology and Intensive Care, University Hospitals of Geneva and University of Geneva, Geneva, Switzerland; 5 Unit of Development and Research in Medical Education, Faculty of Medicine, University of Geneva, Geneva, Switzerland; 6 Division of General Internal Medicine, Department of General Internal Medicine, Geriatrics and Rehabilitation, University Hospitals of Geneva, Geneva, Switzerland; 7 Division of Clinical Epidemiology, University Hospitals of Geneva, Geneva, Switzerland

**Keywords:** Medical decision making, Collaboration, Roles, Internal medicine, Intensive care

## Abstract

**Background:**

Intensive care Unit (ICU) admission decisions involve collaboration between internists and intensivists. Clear perception of each other’s roles is a prerequisite for good collaboration. The objective was to explore how internists and intensivists perceive their roles during admission decisions.

**Methods:**

Individual in-depth interviews with 12 intensivists and 12 internists working at a Swiss teaching hospital. Interviews were analyzed using a thematic approach.

**Results:**

Roles could be divided into practical roles and identity roles. Internist and intensivists had the same perception of each other’s practical roles. Internists’ practical roles were: recognizing signs of severity when the patient becomes acutely ill, calling the intensivist at the right moment, having the relevant information about the patient and having determined the goals of care. Intensivists’ practical roles were: assessing the patient on the ward, giving expert advice, making quick decisions, managing access to the ICU, having the final decision power and, sometimes, deciding whether or not to limit treatment. In complex situations, perceived flaws in performing practical roles could create tensions between the doctors.

Intensivists’ identity roles included those of leader, gatekeeper, life-death decision maker, and supporting colleague doctors (consultant, senior and helper). These roles could be perceived as emotionally burdensome. Internists’ identity roles were those of leader and partner.

**Conclusions:**

Despite a common perception of each other’s practical roles, tensions can arise between internists and intensivists in complex situations of ICU admission decisions. Training in communication skills and interprofessional education interventions aimed at a better understanding of each other roles would improve collaboration.

**Electronic supplementary material:**

The online version of this article (10.1186/s12913-018-3438-6) contains supplementary material, which is available to authorized users.

## Background

Decisions to admit a patient to intensive care (ICU) are often complex. Such decisions are made under time pressure and in a context of fair allocation of resources. Admissions decisions on general internal medicine wards may involve assessment of patients with advanced diseases or elderly patients with multiple comorbidities, for whom the appropriateness of intensive care is uncertain.

ICU admission decisions involve the internist on the ward and the ICU doctor. When a medical in-patient becomes critically ill, the situation is assessed by the internist, who determines whether intensive care is needed and whether the intensivist should be called. The intensivist assesses the patient and discusses the situation with the internist, before the admission decision is made. The two doctors must work together and share their clinical expertise to make the best decision for the patient. Hence, a good collaboration between the two doctors is needed, which supposes availability of clinical information, efficient communication and decision autonomy of collaborators. Apart from these factors, studied in various clinical settings [1–9], clear perception of each other’s roles contributes to effective collaboration [10], as was shown in trauma [5] and internal medicine [11] settings, and among junior doctors and nurses [12]. Clear perception of each other’s roles is far from being obvious however [11] and misconceptions about roles can create frustration [13], insecurity [14], tensions [15] and conflicts [16–19] within teams. Tensions and conflicts can interfere with efficient teamwork [20, 21] and have consequences for patient safety and quality of care [22–25]. The definition of professional roles tends to go beyond the sphere of the strict professional standards and thereby implies individual and collective social identities [26–28]. The purpose of this study was to explore how doctors working on internal medicine wards and intensivists perceive their respective roles during ICU admission decisions and how their perception relates to their experience of such decisions.

## Methods

### Design and setting

This study is a secondary analysis of a qualitative study. The primary analysis concerned the factors influencing the ICU admission decision [29]. The study was conducted at the Geneva University Hospitals, Geneva, Switzerland, a tertiary care hospital with 1741 beds. The study was approved by the Regional Research Ethics Committee of Geneva.

### Participants and data collection

Doctors working in the Divisions of General Internal Medicine and of Intensive Care and directly involved in assessing patients for admission to intensive care were eligible. We included chief residents and residents in internal medicine, as the latter are involved in decisions during night calls. Chief residents are certified in internal medicine in our institution. Eligible ICU doctors were chief residents and attendings. Participants were recruited between March and June 2013. The study was presented at staff meetings in both divisions. Invitations to participate were sent by email to eligible doctors.

We developed an interview guide (see Additional file 1) and pre-tested it with two internists and two intensive care doctors. Qualitative interviews were conducted by a medical sociologist (SC) with no hierarchical relationship to interviewees. After giving their consent to participate, interviewees were asked to reflect on their experience of two significant intensive care admission decisions involving a patient hospitalized in the Division of Internal Medicine. The main objective of the study was to determine the factors facilitating or hindering admission decisions. We explored doctors’ interactions as a potential factor influencing the decision making. Each participant was asked whether he knew the other doctor, whether the other doctor was senior or junior to himself, and what he expected from the other doctor. Whenever possible, questions were asked when they fitted within the participants’ narratives and not according to a fixed sequence as presented in the interview guide. Interviews lasted 57 min on average (min 26, max 94). They were recorded, transcribed verbatim and anonymised.

### Analysis

Interview transcripts were analysed using a thematic content analysis and following an inductive (data grounded) approach [30, 31]. Four interviews (two with internists, two with intensivists) were independently read by members of the multidisciplinary group (three doctors: ME (internist and palliative care doctor), BR (intensivist), MN (internist); one medical sociologist: SC; one medical anthropologist: PH). The group to identified key themes and emergent ideas related (a) to the internists’ and the intensivists’ roles when assessing a patient for intensive care, and (b) to the participants’ perception of the decision making. We developed an initial list of codes [32]. The first 4 interviews were then independently double-coded by 2 researchers (ME, SC). Coding discrepancies were identified and resolved by consensus. Coded interviews were cross-checked by a third researcher. The list of codes was updated based on these four coded interviews. The remaining interviews were then coded by one researcher (ME or SC) and cross-checked by two researchers (ME or SC, and BR or PH or MN), following guidelines on qualitative analysis [33, 34]. Every coding discrepancy was identified and resolved through discussion (ME, SC). New codes were continuously created until saturation. For each new code identified during the coding of interviews, previous interviews were recoded. Codes were then organised into descriptive clusters according to their relatedness in content (e.g. “intensivist as a leader”, “internist has the relevant information about patient”). Coding and analysis were conducted using Atlas.ti Scientific Software Development (Version 7.0.71).

## Results

### Participants

Twelve internal medicine doctors (8 chief residents and 4 residents, hereafter internists) and 12 intensivists (7 chief residents and 5 senior doctors) were interviewed. Most participants were male, with a mean age of 33 and 42 years, respectively (Table 1). Intensivists were more experienced than internists. Among internists, three had never worked in an ICU.

**Table 1:** Characteristics of interviewees

	Internists	Intensivists
	*N* = 12	*N* = 12
Sex (female:male)	3:9	4:8
Age (years), median (range)	34 (27–44)	44 (30–51)
Number of years since graduation, median (range)	7 (3–11)	18 (6–24)
Number of years in the current professional position, median (range)	2 (0–6)	6 (1–11)

### Perceptions of roles during ICU admission decisions

Roles described by the doctors could be divided into practical roles and identity roles. Practical roles concerned their clinical tasks oriented toward concrete actions and decisions. Doctors described these roles factually without any particular emotion. Doctors also described clinical tasks which they identified as constitutive of their professional identity. These tasks were coloured by moral considerations and carried an emotional load. We defined them as identity roles.

#### Practical roles

Practical roles of internists were: recognizing signs of severity when the patient becomes acutely ill, calling the intensivist at the right moment (i.e., not too early and not too late), having the relevant information about the patient (e.g., severity of the underlying disease, patient preferences) and having determined the goals of care (Table 2). Internists and intensivists had the same perception of the internists’ practical roles. One practical role was mentioned by intensivists only: they expected the internists to care for the patient until their arrival on the ward.

**Table 2:** Internists’ practical roles in decisions of admission to intensive care

Roles	Quotes
Recognizes signs of severity	First, [*it’s important to*] identify the early signs [*of a problem*] so that the transfer goes smoothly and things can be managed without all the agitation around reanimation and intubation in an extreme emergency. (Med 07)
Calls at the right moment	We have the impression that things are gradually deteriorating and we call the ICU a bit before things become a big catastrophe. (Med 12)
Has the relevant info about the patient	Before calling the ICU, we have to know the case well. That’s why we don’t call right away. Even if the nurses pressure us and say “you need to call the ICU”, we say “No, no, no, wait. Before calling I need to read the patient’s file”. (Med 02)
Determines the goals of care	It’s important to recognize those situations needing palliative care et not just therapeutic treatments. We have to change the conversation, explain to the patient how things are changing, his risk of dying, and accompany him, make him understand that he is in a different situation now, that he has to envision other possibilities, so that he can prepare and organize. (Med 04)
Continues care until arrival of ICU Dr	Having already initiated certain treatments, these internists know how to do it while waiting for the ICU consultant to arrive. (ICU 11)

The intensivists’ practical roles were: assessing the patient on the ward, giving expert advice, making quick decisions, managing access to the ICU, having the final decision power and, in some cases, deciding whether or not to limit treatment intensity (Table 3). Internists and intensivists had the same perception of the intensivists’ practical roles. One practical role was mentioned by internists only: they expected the intensivists to provide care quickly.

**Table 3:** Intensivists’ practical roles in decisions of admission to intensive care

Roles	Quotes
Makes quick decisions	We decide very quickly and accompany them very quickly. (ICU 04)
Provides care quickly	There can be really urgent situations where the patient risks dying if they don’t intervene quickly with the means we don’t have on the ward. In those situations, we expect [*the ICU*] to come quickly and somehow save the patient, because he’s slipping away from us. (Med 03)
Assesses the patient	Anytime we receive a consultation request, whether it’s for an ICU admission or ends in a refusal, we systematically go to the ward to see the patient. (ICU 01)
Gives expert advice	We have to agree that the treatment plan is technically feasible, so that’s our decision. (ICU 05)
Manages access to ICU	When I say that we have requirements, it’s that we can’t take everyone in the ICU, patients need to have a reason to be here, they have to fulfill criteria for intensive care [...] we can’t take everyone just to help out, it’s not possible. (ICU 03)
Has the final decision power	He [*the internist*] wants to transfer the patient, but we decide if he’s transferred or not. (ICU 10)
Decides whether or not to limit treatment intensity	Every day, we have to decide whether or not to save patients (ICU 04)

#### Identity roles

We identified two types of identity roles: identity roles related to medical decisions and identity roles related to supporting colleague doctors.

The intensivists’ identity roles related to medical decisions were: gatekeeper, life and death decision maker, and leader (Table 4). The identity roles related to supporting were senior, consultant and helper. The internists’ identity roles related to decisions only: leader and partner (Table 5).

**Table 4:** Identity roles of intensivists in decisions of admission to intensive care

Type of identity role	Identity roles	Quotes
Decisions	Gatekeeper	Unfortunately, ICU admission decisions always have serious consequences: it’s important to provide benefit to the patient, avoid harming the patient, and it must also be a just decision for society. (ICU 12)
	Life and death decision-maker	We are more or less capable of making life or death decisions quickly. If we choose [Intensive Care], it’s because we can tolerate it [to make life-death decisions quickly]. We’ve been selected. It’s a Darwinian selection. Those that can’t tolerate it go elsewhere. (ICU 05) The ICU doctor is like an angel of death [...] [who makes] life and death decisions...that is, to stop treatment or not, to consider resuscitation or not. (ICU 06)
	Leader	Often, we sort out the emergencies or the degree of seriousness or worry regarding the patient, and we do it often for our colleagues. It’s part of our job, it’s normal. [...] When we’re called to make such decisions, they usually listen to us and it’s rare really to encounter any opposition. We are often the decision leaders (ICU 03)
Support	Consultant	We’re not going to take their place, we indicate the options to explore, and then possibly afterwards the doctor calls us back [...] because the therapeutic alliance is with the ward doctor. We intervene only as a consultant. (ICU 11)
	Senior	Our role is obviously to be there for the patient, but also maybe to train our colleagues, especially young colleagues working in the emergency department. That’s part of our role, really…we need to be attentive to their panic or worry. If we show up and say “no, it’s not serious” at least the resident is reassured and we won’t have come for nothing because they’ll be able to calmly take care of their other patients. (ICU 03)
	Helper	We’re really, in my opinion, one of the services that helps the most, as often as possible, those colleagues struggling with patients who are on the knife’s edge. (ICU 01)

**Table 5:** Identity roles of internists in decisions of admission to intensive care

Type of identity role	Identity roles	Quotes
Decisions	Leader	What I need is for him [*the intensivist*] to tell me when he can admit the patient…he can of course give his opinion, and if he doesn’t agree to take the patient he has to convince me and my superior why he doesn’t want to. Either he changes his mind, or we change our minds. But often, particularly if we have some experience and if we stick with strict criteria, things are clear. (Med 09)
	Partner	It’s true that the discussion is important, to be sure that the ICU consultant has understood things well. On the ward, we see patients more often, every day, and we have maybe a better sense of what the patient wants than the head of the ICU who arrives and has to get an idea in 15 min. That’s why we’re really complementary. (Med 03)

Some practical roles contributed to the doctors’ professional identity. Intensivists explained that they managed access to the ICU (practical role), but they also felt that it was part of their job to be the gatekeepers of the ICU. Being a gatekeeper implied a sense of moral responsibility in allocating health care resources appropriately. This role was rewarding for intensivists. It was also demanding: to refuse admission to the ICU had potential life-threatening consequences for the patient; it might also cause additional work, for which the resources on the ward were sometimes limited.

In some situations, intensivists decide whether or not to limit treatment (practical role), a decision which they felt had life and death consequences for the patient. This task also contributed to their professional identity. Some intensivists explained they made life-death decisions daily in the ICU, so they felt they were the experts within the hospital. On the one hand, they thought it natural to assume this task for a patient on the ward. On the other hand, some were irritated by their perception that internists sometimes handed the decision over to them. This role was also described as burdensome because life-death decisions had to be made quickly, and sometimes without having all the relevant information. It made intensivists think that one had to “be made for” this medical specialty.

Intensivists saw themselves as leaders of the decision process. It implied assessing the acutely ill patient, then confirming or not the goals of care determined by the internists, and deciding on the intensive care admission accordingly. Intensivists generally played the role of leader in particular situations, for examples when the relevant information was not available or when their determination of the patient’s goals of care was inconsistent with the code status in the patient’s medical file.

Intensivists perceived themselves as senior when they were called by less experienced internists. Being a senior not only meant having more expertise, but also acting as a mentor by providing emotional reassurance, help or teaching (e.g. about the benefits and limitations of intensive care for a given patient). Intensivists felt ready but sometimes also annoyed to act as seniors. They felt it was their job to support less experienced internists when a patient became acutely ill. However, intensivists also perceived this role belonged to senior internists, and it was especially unwelcome when the workload in the ICU was heavy.

Intensivists pointed out that their role was also to be a consultant. As a consultant, they were not supposed to get involved in all the aspects of the decision making process (e.g., discussion with patients or with families, determination of the goals of care). Their role consisted in giving expert advice, like suggesting additional diagnostic hypotheses, or further discussing patient preferences.

Finally, intensivists explained that they were helper to their colleagues: they helped assess and manage critically ill patients on the wards, and they sometimes relieved the healthcare professionals on the ward by admitting an acutely ill patient who had no clear need for intensive care. Intensivists felt that their commitment was not always properly acknowledged.

Internists felt they had a role of leader in the ICU admission decision because they provided the necessary information about the patient. They explained it was part of their job to determine the patient’s goals of care, a task which gave them key knowledge for making admission decisions .

Internists also perceived themselves as partners of the decision. Internists agreed that intensivists had the final decision power, but emphasized that admission decisions should result from a collaborative decision making process.

### Sources of tension related to doctors’ practical roles

Participants were generally satisfied with the way their colleague doctors performed their practical roles. Complaints occurred in relation with complex situations, when perceived flaws in performing practical tasks were more likely to increase the doctors’ uncertainty about the appropriateness of the final decision, admission or no admission. Tensions between doctors could then arise. We identified three types of situations when tensions were related to practical roles.

#### Providing relevant information

Both internists and intensivists reported that having the relevant information was particularly important for the admission decision in complex situations. Intensivists complained that they did not always receive it. This typically occurred when the requesting internist did not know the patient well, as in on-call situations (nights and week-ends), or when patients had been transferred to an intermediary care unit. Intensivists were, however, aware of this structural constraint and showed understanding towards the internists:

A young internist who deals with I don’t know how many internal medicine wards won’t necessarily know the advance healthcare directives and co-morbidities of all patients. (ICU 08).

Internists complained that the intensivists were not willing to listen to and discuss the information, and that they expected to be convinced of the appropriateness of an ICU admission. Internists felt that a slight imperfection in imparting the information might prompt the intensivists to use their decision power and refuse admission of a patient. Some intensivists acknowledged this behaviour:

It is enough that he [the internist] uses the wrong or unconvincing words for us, who manage the shortage of beds, to say: “Wait, he doesn’t have a clear indication for admission”. (ICU 05).

Both internists and intensivists compared their interaction with a hard bargain, which internists sometimes found burdensome:

What’s tiring is presenting patients to the ICU and having to justify why, and having admission criteria that are a bit rigid. There’s a sort of fighting, which seems a bit like selling. (Med 06).

There’s the marketing aspect, there’s a whole sales process. (ICU 05).

#### Determining the goals of care: Choosing comfort care is difficult

Determining the goals of care involves the internists’ role of discussing (a) with the patient before the acute event about his or her preferences (including the possibility of ICU admission), and (b) with the intensivist during the acute event, i.e. discussing the benefit of intensive care in the light of the goals of care previously determined. We observed discordant perceptions between internists and intensivists about decisions involving changing the goals of care into comfort care.

Internists reported that they routinely discussed preferences and goals of care with patients and then made decisions about code status, including comfort care. They acknowledged however that decisions of comfort care, or no cardiopulmonary resuscitation were sometimes difficult to make.

That’s also part of our specialty [...] to recognize situations where we need to stop, where we should leave the patient in peace, knowing that we have nothing better to offer. (Med 04).

It’s not obvious to specify NTBR [Not To Be Reanimated]. I find it hard sometimes, too, but it’s important. (Med 08).

Some intensivists reported a feeling of annoyance. They reproached the internists for a lack of anticipation in discussing goals of care with seriously ill and elderly patients.

It seems like there’s no anticipation, that the residents wait for things to become serious or for an emergency before asking “At 87, do you want to be resuscitated? Do you want to be intubated? Do you have advance healthcare directives? Have you discussed this with your children?”. (ICU 09).

Intensivists also supposed that the internists sometimes found it difficult to take their responsibility and limit treatment intensity:

I think it’s a recurring problem that we have with internal medicine, who puts the onus of decision-making on the ICU doctor to pursue intensive treatment or not, because the residents don’t feel able to make this decision. (ICU 11).

#### Giving expert advice: Misunderstanding about expected role

Internists explained that they sometimes called the ICU doctors as consultants, simply to discuss a complex situation. Intensivists usually assumed that the internists wanted the patient to be admitted to intensive care. Thus, they behaved as gatekeepers. Such mismatched expectations could cause tensions.

I think it can be important to discuss a case, to have varying viewpoints...And not because of indecision, but just because it’s a situation where there may be different viewpoints. [...] What was a bit disturbing in this particular situation was the lack of openness on the part of the ICU to discuss. They always have the impression we’re asking them to decide for us, which was not the case here, we just wanted to discuss the case. (Med 06).

## Discussion

In this study, we described the roles of internists and intensivists during decisions of admission to intensive care. We found two types of roles: roles related to practical clinical tasks and roles related to activities constitutive of doctors’ professional identity. This distinction between practical and identity roles is in line with organisational psychology [35] and means that the definition of professional roles exceeds the practical standards and includes identity components [26–28]. We observed that identity roles of internists and intensivists were coloured by moral considerations and emotional load, a result in line with studies examining the building process of work-related identity [26, 36]. Doctors valued the identity roles they described (e.g. leader, life-death decision maker, senior). This finding is in line with the social identity approach [37] and has been observed in other healthcare settings [11, 38, 39]. The social identity approach [37] emphasizes that individuals try to achieve and maintain positive social identity, i.e. concepts of themselves that favour self-esteem and distinctiveness.

Interestingly, intensivists’ identity roles were not only based on activities related to decisions of ICU admission, but also on activities supporting the clinical work of their younger internist colleagues, especially during night calls. This contrasts with the sometimes dismissive attitude previously reported [40, 41]. Intensivists however were ambivalent regarding their role as senior. To work as a benevolent and supporting experienced doctor was gratifying, but intensivists also expressed frustration when they had to interact with junior doctors and not with their senior internist counterparts, a situation that happens mostly at night.

Some roles were sometimes perceived as burdensome. To determine the goals of care for complex patients or to act as a gatekeeper could be a source of stress. Although doctors acknowledged that comfort care was most appropriate for some critically ill patients, the feeling of not providing the best chances of survival was sometimes emotionally challenging. Ultimately, such stressful experiences may be identity-relevant [42]: they reflect the necessity for doctors to balance survival benefit against other goals in medicine, such as symptom control, and to make decisions which might generate regrets [43]. Stressful experiences can also strengthen professional identity [44]. In our institution for example, if a patient is not admitted to intensive care, the intensivist is no longer involved in his care on the ward. Depending on the patient’s condition, the internist will then have to provide palliative and sometimes end of life care. The internist can have support from the palliative care team, who work as consultants, assessing the patient, giving treatment advice and following the evolution of the patient’s condition. Palliative care services however are available on week days only.

Some intensivists described themselves as life and death decision-makers, a role contributing to their professional identity. This role was described as burdensome, especially in complex situations. Complex patient care-related situations have been associated with doctors’ moral distress [45, 46] and sleep disorders [47]. Such data echo with the high prevalence of burn out [48, 49] and depression [50] found in intensivists. The role of life and death decision-maker however was also mentioned positively, as a marker of special competencies and engrained abilities. Such a perception on the intensivists’ part may interfere with an open exchange of views and collaborative decision making with the internists during admission decisions [29, 40, 41].

Although internists and intensivists had, overall, the same perceptions of each other’s practical roles and were generally satisfied with the way they were enacted, complex clinical situations put a strain on the admission process and were likely to cause tensions between the doctors. Previous studies have shown that tensions and conflicts can jeopardize patient safety and quality of care [24, 51]. With the increase in life expectancy [52, 53] and in chronic conditions in the general population [54], the number of complex clinical situations will increase, underscoring the need to effectively resolve any conflicts between internists and intensivists. Potential for tension was increased in our study by the difference in experience between some internists and ICU doctors, e.g. a resident in internal medicine and an attending intensivist. Intensivists in particular felt that the internists might not be up to their clinical tasks, such as discussing goals of care with patients, and expressed frustration as they sometimes felt they had to compensate for the internists’ lack of experience. Not meeting the other doctor’s expectations about completion of practical roles is likely to have an impact on doctor inter-relationship. Quality of interactions has been shown to be an important determinant of doctors’ satisfaction with admission decision making and might influence the care of future patients [29].

Our findings put in light the internists’ key role in admission decisions. Both intensivists’ and internists’ narratives showed that the whole process depended on the internists’ abilities to perform practical tasks satisfactorily. In particular, internists must collect the relevant information and impart it to the intensivists in a coherent and structured way. The view of medical teams based on common purpose, effective communication, good cohesion and mutual respect [55] was observed in our findings, but we also observed that internists’ and intensivists’ interactions were made of negotiations and tensions. Doctors portrayed some admission decisions as a trade or bargain. The process of trade was also found in the daily teamwork of intensive care units and described as a catalyst for tension within the team [56].

One source of tensions was related to misunderstanding about expected roles, when internists needed expert advice and intensivists acted as ICU gatekeepers. Role misunderstanding can negatively impact on communication and collaboration between and within health care disciplines [57] and thus may have negative consequences on the quality of care.

### Limitations and strengths

Our study has limitations. It was conducted in a single academic tertiary hospital, limiting the possibility to generalise our findings to other settings. The study addressed ICU admission decisions from internal medicine wards, excluding other situations of admission decisions from the operating rooms, surgical units or the emergency department [41]. The focus was on more complex situations and we may have not identified significant roles related to straightforward admission decisions. Of note, we explored doctors’ perceptions about the process of admission decisions. We did not explore how the internists determine goals of care and decide on treatment limitations for patients, an important daily clinical task the intensivists in our study were not fully aware of. Among strengths, we limited socially desirable responses by conducting interviews with a medical sociologist. Data analysis was conducted by two researchers (ME, SC) to reduce the researcher bias (i.e., the researcher is both the data collector and data analyst) [58]. It was cross-checked by clinicians (MN, BR) and a medical anthropologist (PH) to strenghten its validity.

## Conclusions

Despite a common perception of each other’s practical roles, tensions can arise between internists and intensivists in complex situations of ICU admission decisions. The conditions for good collaboration are not limited to the sphere of practical roles, but are likely to include a better recognition of doctors’ identity roles. Training in communication skills and interprofessional education interventions aimed at a better understanding of each other roles would be a way to improve collaboration and potentially foster quality of care for critically ill patients.

## Additional file

Additional file 1: Interview guide. (DOCX 54 kb)

## Abbreviations

ICU: Intensive care unit
Med: Internal medicine
