# Supplementary material for: Internists’ and intensivists’ roles in intensive care admission decisions: a qualitative study
Source: BMC Health Serv Res. 2018 Aug 8;18:620. doi: 10.1186/s12913-018-3438-6 (PMC6083517; doi:10.1186/s12913-018-3438-6)
Supplement: Supplementary file 1 — Interview guide. (DOCX 54 kb) [file 12913_2018_3438_MOESM1_ESM.docx]

**Additional file 1**

**Interview guide**

Please tell me about the situation you have chosen to discuss today*.*

- Patient characteristics: age, illness, attitude/code status, indications for requesting ICU admission
- Context: when/where it occurred, who was involved (other doctors, family members)
- Interactions between the Internal medicine and Intensive care doctors:
  - Did the SI physician come to see the patient?
  - Did you know the other physician?
  - Was the other physician senior or junior to you?
  - What did you expect from the physician?
  - Was the patient admitted to intensive care unit?
- In your opinion, what facilitated or complexified the decision-making process in this situation?

(*Above* *questions were repeated with a second situation chosen by interviewees)*

- (*If the respondent discussed two situations*): Could you compare the two situations?
- In your opinion, what is an ideal ICU admission decision-making process?
